# A Transition State Resonance Radically Reshapes Angular Distributions of the *F* + *H*_2_ → *FH*(*v*_f_ = 3) + *H* Reaction in the 62–102 meV Energy Range

**DOI:** 10.1021/acsphyschemau.4c00096

**Published:** 2025-02-04

**Authors:** Dmitri Sokolovski, Dario De Fazio, Elena Akhmatskaya

**Affiliations:** † Departmento de Química-Física Química-Física, Universidad del País Vasco, UPV/EHU, 48940 Leioa, Spain; ‡ IKERBASQUE, Basque Foundation for Science, Plaza Euskadi 5, 48009 Bilbao, Spain; § EHU Quantum Center, Universidad del País Vasco, UPV/EHU, 48940 Leioa, Spain; ∥ Istituto di Struttura della Materia-Consiglio Nazionale delle Ricerche, 00016 Roma, Italy; ⊥ Basque Center for Applied Mathematics (BCAM), Alameda de Mazarredo 14, 48009 Bilbao, Spain

**Keywords:** physical chemistry, chemical reactions, differential cross sections, scattering resonances, complex angular momentum analysis

## Abstract

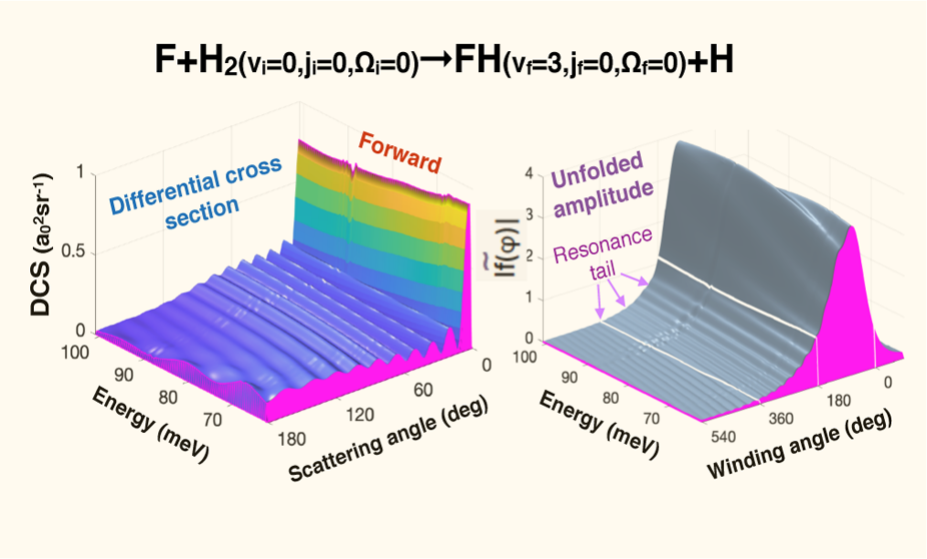

Reactive angular distributions of the benchmark *F* + *H*_2_(*v*_i_ = 0) → *FH*(*v*_f_ = 3) + *H* reaction show unusual propensity toward small scattering angles, a subject of a long debate in the literature. We use Regge trajectories to quantify the resonance contributions to state-to-state differential cross sections. Conversion to complex energy poles allows us to attribute the effect almost exclusively to a transition state resonance, long known to exist in the *F* + *H*_2_ system and its isotopic variant *F* + *HD*. For our detailed analysis of angular scattering we employ the package DCS_Regge, recently developed for the purpose [AkhmatskayaE.; SokolovskiD.Comput. Phys. Commun.2022, 277, 108370].

Reactive differential cross sections (DCS) are known to be sensitive to the details of reaction mechanism and are, therefore, a source of useful information. A simple picture of a collision between an atom and a diatomic molecule is as follows (see, e.g., in ref (1)). In atom–diatom reaction, transfer of an atom is more likely when the distance between the collision partners is short, i.e., for small angular momenta, or small impact parameters. With the forces acting between the atoms being of predominantly repulsive nature, a rapid encounter between the reactants is likely to favor large reactive scattering angles θ_R_. Such a direct mechanism can, therefore, be expected to produce a backward-peaked reactive DCS, rapidly decreasing as θ_R_ tends to 0.

This is, however, not what one observes for certain transitions of the benchmark reaction *F* + *H*_2_ → *FH* + *H*.^2−4^ Contrary to the above expectation, the DCS for rovibrational manifolds *v*_i_ = 0, *j*_i_ = 0 → *v*_f_ = 3, *j*_f_ = 0, 1, 2 exhibit a high forward (θ_R_ = 0) peak, followed by pronounced oscillations which affect the entire angular range 0° ≤ θ_R_ ≤ 180°. The question whether resonances can lead to observable effects in the *F* + *H*_2_ reaction has long been debated in the literature,^4^ with diverse views expressed by the authors of refs (5−11) and (3,12−16). Thus, in ref (3) it was demonstrated that the forward peak observed for *FH*(*v*_f_ = 2) products was indeed caused by Feshbach resonances. The authors of ref (9) drew a distinction between *v*_f_ = 2 and *v*_f_ = 3 cases, and argued that for *v*_f_ = 3 the enhancement of the small angle scattering was a result of dynamically different slow-down mechanism. This view was supported in ref (11), although without a direct reference to metastable states, or poles of the *S*-matrix element. Further readings on the subject can be found in a recent review^17^ and references therein.

The question is best settled by an analysis, capable of both quantifying an effect and unambiguously linking it to a resonance known to exist for the *F* + *H*_2_ system. Recently, a study of three *F* + *H*_2_ → *FH* + *H* zero-helicity transitions, Ω_i_ = Ω_f_ = 0, at a translational energy of 62.09 meV, related the unusual behavior of the state-to-state reactive DCS to the presence of a single Regge pole.^18^ The purpose of this paper is to confirm this hypothesis and complete the analysis of ref (18) by extending it to a broader energy range 62.09–101.67 meV. We obtain the relevant Regge trajectories and, by converting Regge poles into poles in the complex energy plane, attribute the effect to just one transition state resonance,^13^ known to exist for the *F* + *H*_2_ system and its isotopic variant *F* + *HD* (see, for example, refs (19−21)).

## I Methods

For our analysis we will rely on a methodology somewhat different from those used in.^18^ It was recently implemented in the software DCS_Regge,^22^ now available in the public domain: https://data.mendeley.com/datasets/gf4gm82n6m/1. The method is discussed in detail in,^22^ and here we only repeat what is necessary for the present narrative. A differential cross section (DCS), σ_ν_f_←ν_i__, at an angle θ_R_ and an energy *E* is given by an absolute square of the scattering amplitude,
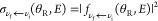
1where the composite indexes ν_i_ = (*v*_i_, *j*_i_, Ω_i_) and ν_f_ = (*v*_f_, *j*_f_, Ω_f_) include the initial and final vibrational (*v*), rotational (*j*) and helicity (Ω) quantum numbers of the system. In the zero-helicity case, Ω_i_ = Ω_f_ = 0, the amplitude is given by a simple partial wave sum
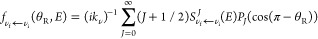
2where *J* is the total angular momentum quantum number, *S*_ν_f_←ν_i__^*J*^ is a body-fixed scattering matrix element, *k*_ν_ ≡ *k*_*v*_i_, *j*_i__ is the initial translational wave vector of the reactants, and *P*_*J*_(•) is Legendre polynomial (see, e.g., ref (23)). In practice, the sum is terminated at some *J* = *J*_max_ ≫ 1. This inequality holds under the semiclassical condition, assumed throughout the rest of the paper.

For a chosen state-to-state transition, the code DCS_Regge evaluates two “unfolded” amplitudes (the usual shorthand notation λ ≡ *J* + 1/2 is used below)
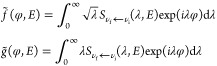
3Both are functions of the newly introduced angular variable φ, which varies between −∞ and ∞. The function *S*_ν_f_←ν_i__(λ, *E*) in (eq 3), such that *S*_ν_f_←ν_i__(*J* + 1/2, *E*) = *S*_ν_f_←ν_i__^*J*^(*E*), *J* = 0, 1, ··· *J*_max_, is the analytic continuation of the *S*-matrix element into the complex angular momentum (CAM) plane, achieved by means of Padé approximation (more details are given in ref (24)). The scattering amplitude (eq 2) for π/*J*_max_ ≲ θ_R_ ≲ π – π/*J*_max_ can now be obtained by “folding back” the amplitudes (eq 3)^22^
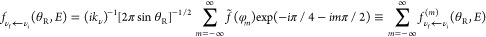
4where
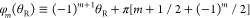
5For θ_R_ = 0, and θ_R_ = π one has
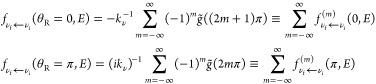
6The rationale behind (eq 4) becomes clear by considering the classical limit of atom–diatom reaction A + BC → AB + C. The variable of interest is the winding angle φ, i.e., the angle swept in the course of the collision by projection of the Jacobi vector **R**_A←BC_, drawn from the center of mass of the BC pair to the atom A, onto the plane perpendicular to the total angular momentum **J** (see Supporting Information A). One can attempt to achieve a semiclassical description by ascribing probability amplitudes to the reactive trajectories leading to all winding angles φ_*m*_(θ_R_), consistent with the scattering angle θ_R_ [cf. (eq 5)], and adding up the amplitudes in accordance with the basic rule of quantum mechanics. The correct formula 4 contains, however, additional phase factors exp(−*i*π/4 – *imπ*/2), and fails for both small and large scattering angles. The reason for this is the coalescence of winding angles π – θ_R_ + 2*mπ* and π + θ_R_ + 2*mπ* as θ_R_ → 0, and of π – θ_R_ + 2*mπ*, and π + θ_R_ + 2(*m* – 1) π as θ_R_ → π. With the two angles no longer distinguishable, (eq 4) must be replaced by one of the (eq 6). (A detailed discussion of (eq 6) can be found in ref (25)).

The analysis proceeds by examining the shapes of *f̃* (φ, *E*) and *g̃*(φ, *E*). For a direct reaction one can expect *f̃*(φ, *E*) essentially limited to the interval 0 ≤ φ ≤ π, negligible for φ ≈ 0, considerable for φ ≈ π and, perhaps, having a small extension into the φ < 0 region due to purely quantum effects. For a reaction, passing through formation of one or several intermediate rotating complexes, *f̃*(φ, *E*) is likely to extend also into the φ ≥ π zone. This extension is expected to take the form of one or several “exponential tails”, which resonance CAM (Regge) poles at *J*_*n*_, *n* = 1, 2···, in the first quadrant of the CAM plane contribute to the integrals in (eq 3)^25^
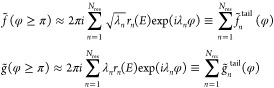
7In (eq 7), *r*_*n*_(*E*) ≡ lim_*J*→*J*_*n*__(*J* – *J*_*n*_) *S*_ν_f_←ν_i__(*E*, *J*) is the residue of the *S*-matrix element at *J* = *J*_*n*_, and the sum is over *N*_res_ “physically significant poles”. The notion is, in this context, self-explanatory. Significant is a pole whose contribution to *f̃*(φ ≥ 0) and *g̃*(φ ≥ 0) is sufficient to cause observable interference effects in the DCS [cf (eq 4)]. Such a pole must lie not too far from the real *J*-axis (or its tail will be too short), and have a sufficiently large residue (or the tail will be too small). Finally, we note that (eq 7) are consistent with a picture of a rotating intermediate triatomic complex which continues to decay into products. The triatomic’s moment of inertia *I* together with Re[*J*_*n*_] determine its angular velocity, ω ≈ Re[*J*_*n*_]/*I*, while 1/Im[*J*_*n*_] yields the typical rotation angle.^22^

Next we check whether for the chosen transitions the unfolded amplitudes in (eq 3) have tails described by (eq 6), and ascribe every found tail to a Regge pole, or poles, of the *S*-matrix element. Importantly, we will relate each CAM pole to its complex energy counterpart, and explain the shape of the DCS in terms of the well-known *F* + *H*_2_ resonances,^5,15^ something not yet done in.^18^

## II Results

As in ref (18), we use the *S*-matrix elements of the title reaction, computed by the hyperspherical method of ref (26) on the Fu-Xu-Zhang (FXZ) potential energy surface (PES).^27^

### II.I (0,0,0) → (3,0,0) Transition

**Figure 1:**
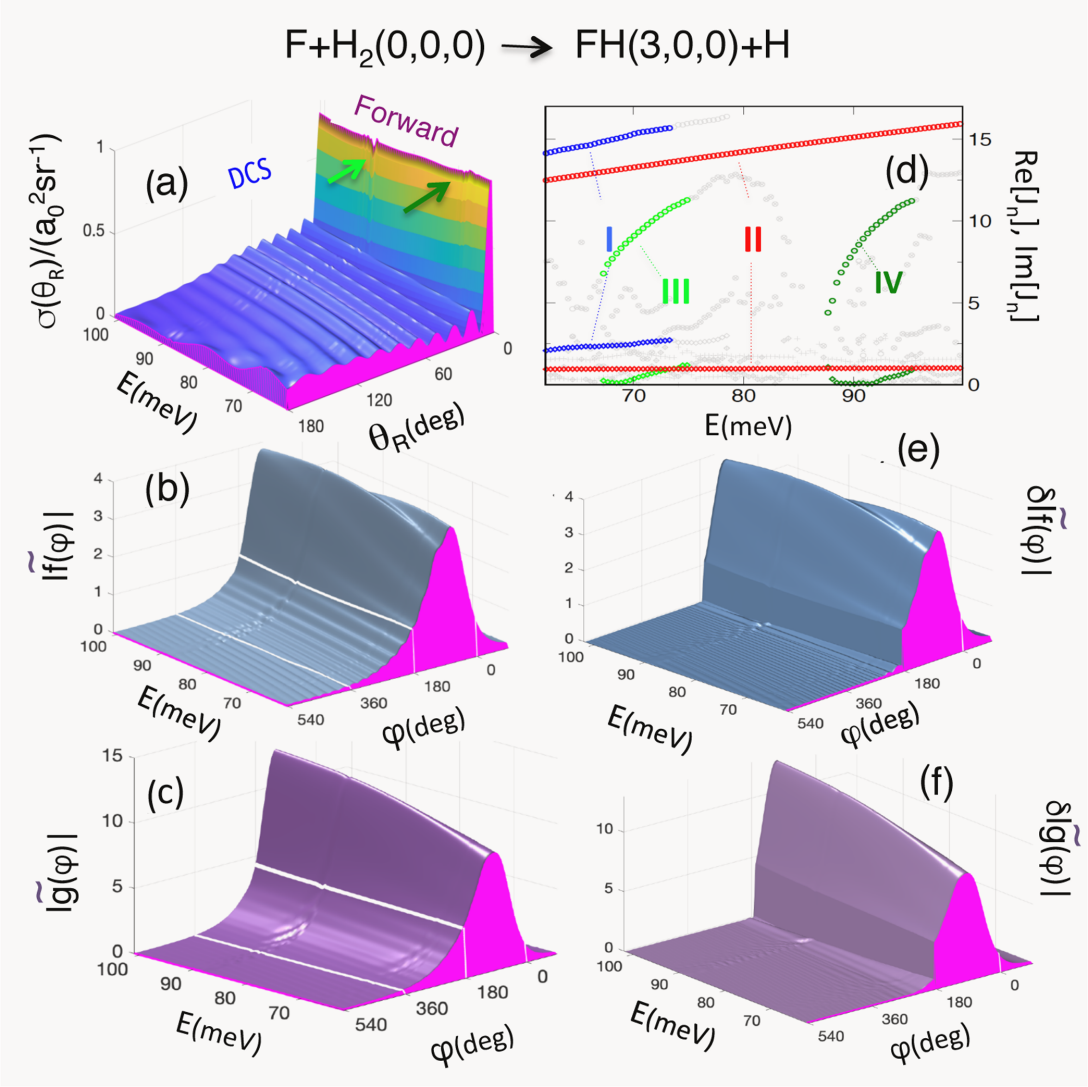
(a) Reactive differential cross section σ_ν_f_←ν_i__(θ_*R*_) for the *v*_i_ = 0, *j*_i_ = 0, Ω_i_ = 0 and *v*_f_ = 3, *j*_f_ = 0, Ω_f_ = 0 vs θ_R_ and *E*. Small patterns which occur in the regions 65–70 meV and 85–90 meV in the entire angular range are indicated by arrows. (b) The modulus of the unfolded amplitude *f̃* vs the winding angle φ and *E*. (c) Similar to (b), but for the unfolded amplitude *g̃*. (d) Real (circles) and imaginary (diamonds) parts of the four Regge trajectories. (e) The difference between |*f̃*(φ)| and |*f̃*_II_^tail^(φ)| [cf. (eqs 3 and 7)] subtracted for φ > 180°. (f) The difference between |*g̃*(φ)| and |*g̃*_II_^tail^(φ)| [cf. (eqs 3 and 7)] subtracted for φ > 180°.

The DCS (eq 1) shown in Figure 1a, exhibits a high forward scattering peak, whose height varies little across the translational energy range 62.09–101.67 meV considered here. There are also regular oscillations observed at all energies in the entire angular range 0 ≤ θ_R_ ≤ 180°. A closer inspection reveals much smaller patterns superimposed on the DCS in the regions 65–71 meV and 85–92 meV (indicated by arrows in Figure 1a). To explain this behavior we employ the DCS_Regge code of ref (22)

Both unfolded amplitudes in (eq 3), *f̃*(φ, *E*) and *g̃*(φ, *E*), shown in Figure 1b,c, respectively, are smooth functions of φ and *E*, and have tails which extend into φ ≥ 180° region and become negligible at φ ∼ 500°. The oscillations in Figure 1a must, therefore, result from interference between the part of *f̃* contained in the region 0 ≤ φ ≤ 180°, and the tail, which we expect to be produced by capture into a metastable state, or states [cf. (eqs 4 and 7)].

To check whether this is the case, we plot positions of the Regge poles in Figure 1d. There are four Regge trajectories, labeled by Roman numerals, *n* = I, II, III, IV. For *E* = 62.09 meV, trajectory II contains the pole at *J* = 12.49 + 0.95*i*, previously found in,^18^ and we expect it to be the most significant of the four. Indeed, in Figure 1e the difference δ|*f̃* (φ, *E*)| ≡ |*f̃* (φ, *E*)| – |*f̃*_II_^tail^ (φ, *E*)|χ(φ – π), where χ(*x*) = 1 for *x* ≥ 0 and 0 otherwise, practically vanishes for φ ≥ 180°, which shows that the pole II is responsible for almost all of *f̃*(φ, *E*) φ ≥ 180° region. The same is true for the difference δ|*g̃*(φ, *E*)| ≡ |*g̃* (φ, *E*)| – |*g̃*_II_^tail^ (φ, *E*)|χ(φ – π), shown in Figure 1f. Thus, there can be little doubt that the tails of both unfolded amplitudes and, therefore, the oscillations in the DCS in Figure 1a, are largely caused by the resonance II [cf. Figure 1f].

**Figure 2:**
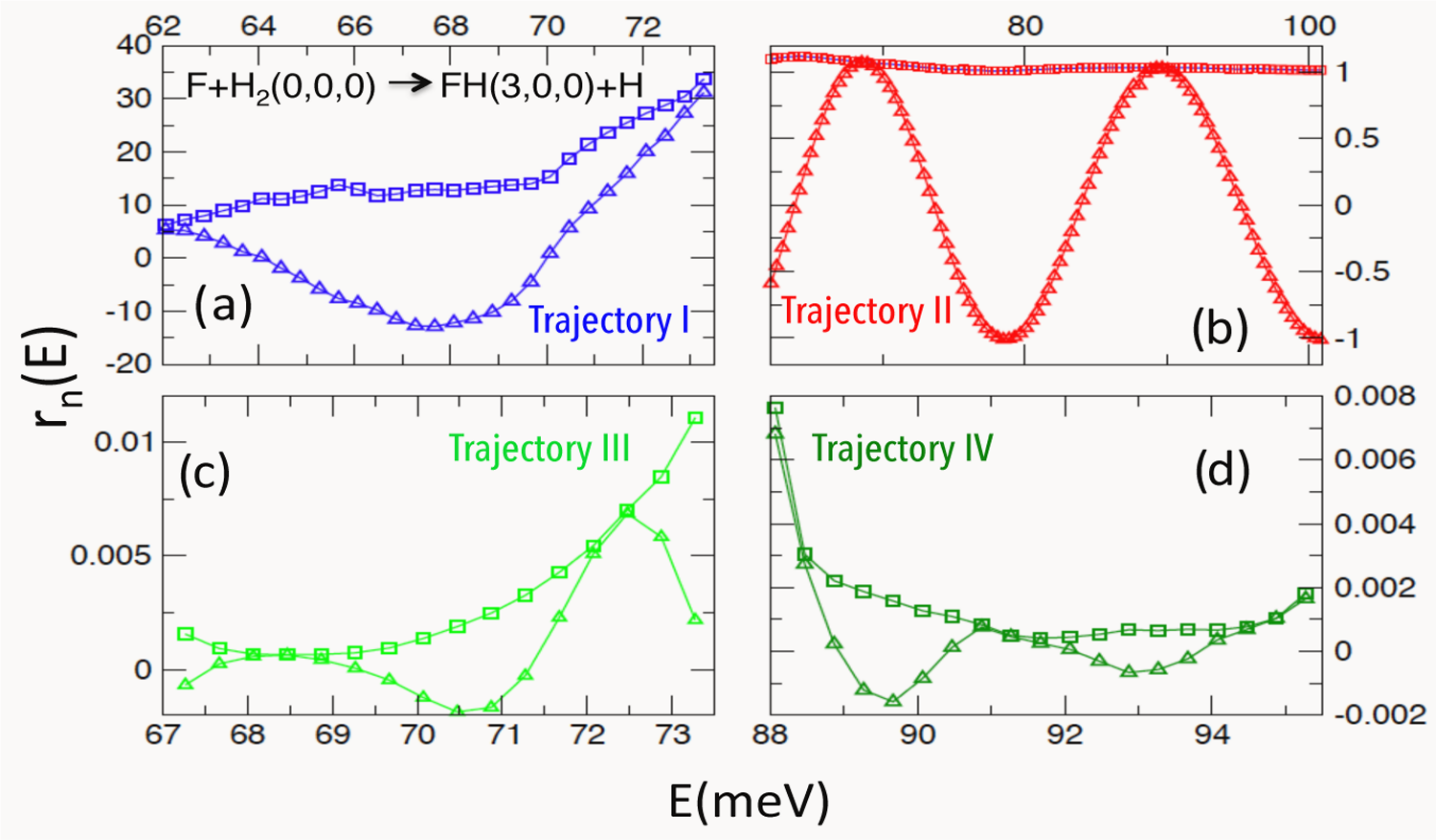
Moduli (squares) and real parts (triangles) of the residues *r*_*n*_(*E*), *n* = I, II, III, IV, for the four Regge trajectories in Figure 1d.

An inspection of the pole positions in Figure 1d and the corresponding residues in Figure 2 explains why other poles can have only minor effect on σ_3,0,0←0,0,0_(θ_R_, *E*). The resonance I (i.e., the Regge pole I) has the largest residue, but also a large imaginary part. For φ ≥ π, its tail, reduced by a factor exp {−Im[*J*_I_]φ}, is both short and small. (It needs, however, to be taken into account when calculating the forward scattering cross section at lower energies, as will be shown shortly.) The residues of the poles III and IV are very small, and even though both resonances are long-lived [cf. Figure 1d], they are accountable only for the small patterns indicated by arrows in Figure 1a.

To quantify the analysis, we consider the energy dependence of the DCS at a chosen scattering angle, first by identifying the important terms in the expansions (eqs 4–6), using, where possible, approximations (7), and comparing the result with the exact DCS.

**Figure 3:**
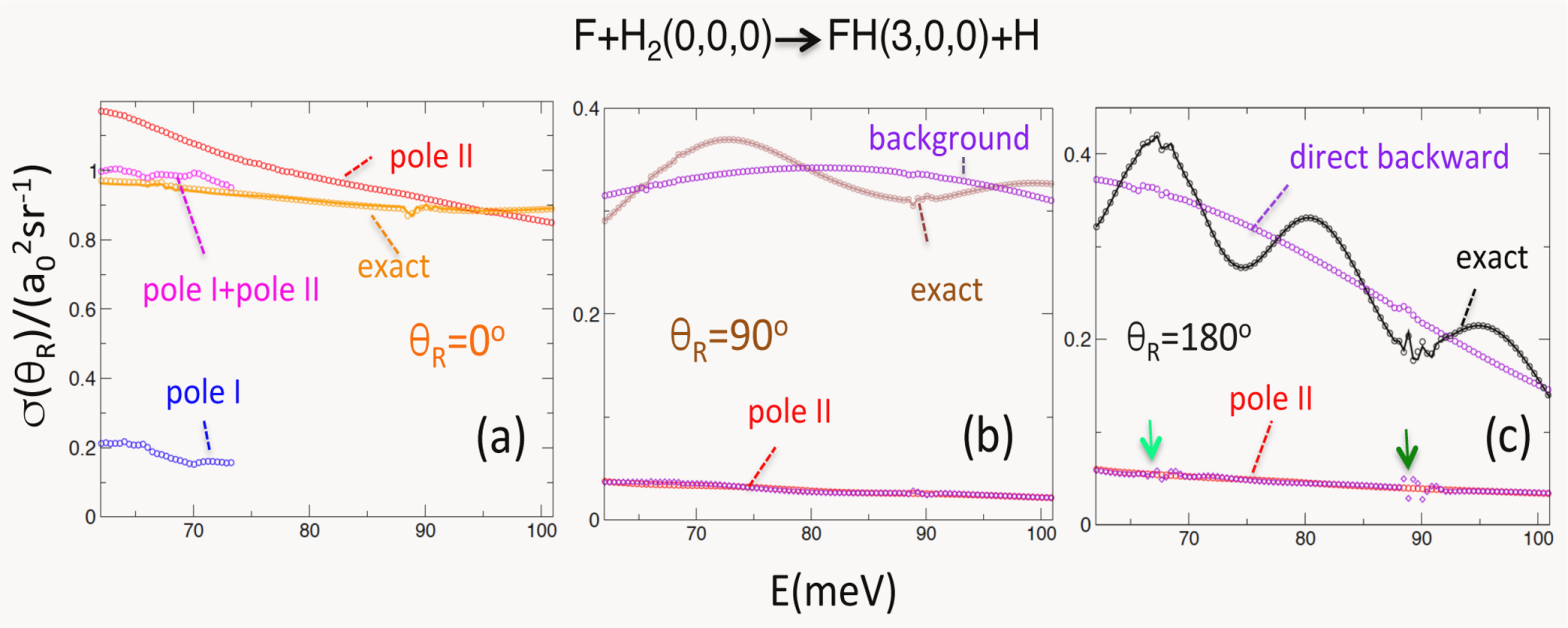
(a) Exact DCS (eq 1) at θ_R_ = 0° (solid orange) and |*f*_ν_f_←ν_i__^(0)^(0, *E*)|^2^ (orange circles). Also shown are the resonance contributions *k*_ν_^–2^ |*g̃*_I_^tail^(π)|^2^ (blue circles), *k*_ν_^–2^|*g̃*_II_^tail^(π)|^2^ (red circles), as well as a coherent sum of the two, *k*_ν_^–2^|*g̃*_I_^tail^ + *g̃*_II_^tail^(π)|^2^ (malva circles). (b) Exact DCS (eq 1) at θ_R_ = 90° (solid brown). Also shown are the background term, |*f*_ν_f_←ν_i__^(−1)^(π/2, *E*) + *f*_ν_f_←ν_i__^(0)^(π/2, *E*)|^2^ (violet circles), |*f*_ν_f_←ν_i__^(1)^(π/2, *E*)|^2^ (violet diamonds), and their coherent sum |∑_*m*=–1_^1^*f*_ν_f_←ν_i__^(*m*)^(π/2, *E*)|^2^ (brown circles). Contribution of the resonance II, |(2π)^−1/2^*k*_ν_^–1^*f̃*_II_^tail^(3π/2) exp (−*iπ*/4)|^2^ (red circles) is in good agreement with |*f*_ν_f_←ν_i__^(1)^π/2, *E*)|^2^. (c) Exact DCS (eq 1) at θ_R_ = 180° (solid black). Also shown are the direct term |*f̃*_ν_f_←ν_i__^(0)^(π, *E*)|^2^ (violet circles), |*f̃*_ν_f_←ν_i__^(1)^(π, *E*)|^2^ (violet diamonds), and their coherent sum |∑_*m* = 0_^1^*f*_ν_f_←ν_i__^(*m*)^(π, *E*)|^2^ (black circles). Contribution of the resonance II, *k*^–2^|*g̃*_II_^tail^(2π)|^2^ (red circles), is in good agreement with |*f̃*_ν_f_←ν_i__^(1)^(π, *E*)|^2^, with small discrepancies noted where the resonances III and IV need to be taken into account (as indicated by the arrows).

**Figure 4:**
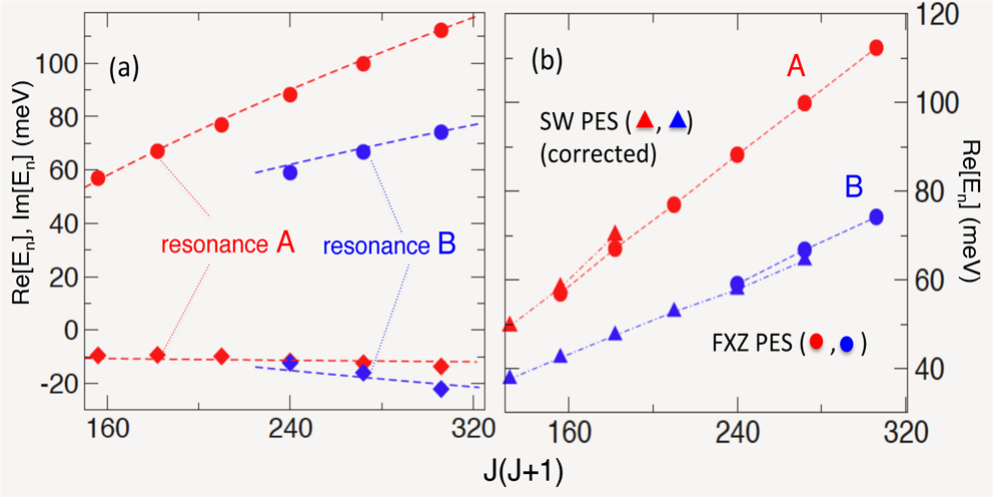
(a) Real (circles) and imaginary (diamonds) parts of the exact complex energy poles, corresponding to the Regge trajectories I (blue) and II (red) in Figure 1d. Also shown by the dashed lines is the approximation (eq 11). The variable *J*(*J* + 1), rather than *J*, is used to facilitate comparison with Figure 9 of ref (15). (b) Comparison between real parts of the CE poles obtained for the FXZ PES (circles, present work), and the corresponding poles for the SW PES (used in ref (15), triangles). The SW results are corrected downward by 13 meV (see Figure S2 of the Supporting Information).

A good approximation to the forward DCS is obtained by taking into account only the zeroth term in (eq 6), *f*_ν_f_←ν_i__^(0)^(0, *E*) (orange circles in Figure 3a). One can try to attribute it to the decay of the recently formed resonance II into the forward direction θ_R_ = 0, *f*_ν_f_←ν_i__(0, *E*) ≈ −*k*_ν_^–1^*g̃*_II_^tail^(π). The result, shown by red circles in Figure 3a, can be improved, by about a fifth (malva circles in Figure 4a), by including also the contribution from the resonance I
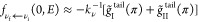
8[We are able to follow Regge trajectory I only until it leaves the region where Padé approximation can be trusted at *E* ≈ 74 meV.^24^ The remaining small discrepancy is attributed to the inaccuracy of the asymptote (eq 7) if the resonance is formed at a large value of *J*(25)].

The sideway DCS at θ_R_ = 90°, shown in Figure 3b (solid brown), is, to an excellent accuracy, a result of interference between the “background” term (violet circles), *f*_ν_f_←ν_i__^(−1)^(−π/2,*E*) + *f*_ν_f_←ν_i__^(0)^(π/2,*E*) and *f*_ν_f_←ν_i__^(1)^(−π/2, *E*) (violet diamonds). The latter term is seen to be a result of the decay of the resonance II (red circles) after **R**_F←HH_ rotates by 3π/2 [cf. (eqs 6 and 7)],
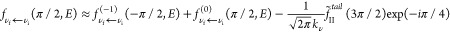
9(Note that DCS_Regge does not distinguish between direct scattering and decay of a resonance for φ lying between 0 and π. A more sophisticated technique is available,^25^ but was deemed too cumbersome to be included into the software.)

Finally, the oscillations of the *backward* DCS (black solid) in Figure 3c are clearly the result of interference between a direct recoil following a head-on collision, and the decay of the resonance II after **R**_F←HH_ completes one full rotation [cf. (eq 7)]
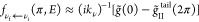
10

### II.II Assignment of Regge Resonances

There are two complementary ways of relating the same resonance phenomenon to a singularity of a scattering matrix element *S*_ν_f_←ν_i__(λ, *E*). Fixing a real value of *J* allows one to look for poles in complex energy (CE) plane, while fixing a value of *E* gives rise to CAM Regge poles. The CE poles, whose relation to the PES is usually well understood (see ref (3,15,16)), are not particularly useful for a quantitative analysis of the integral and differential cross sections, given by sums over angular momentum at a fixed value of *E*. The Regge poles, for their part, are well suited for such an analysis, but can offer little insight into the dynamics on the PES. Fortunately, positions of the poles of one kind can usually be obtained if the positions of poles of the other kind are already known,^28^ so the benefits of both approaches can be combined.

In the present case, the task is especially easy since the pole positions of the resonances I and II are practically linear functions of *E* in their respective energy ranges, *J*_I,II_ ≈ α_I,II_ + β_I_,_II_*E*. Inverting the relation yields the positions of the corresponding CE poles, *E*_I,II_
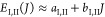
11with the complex constants given by *a*_I,II_ = −α_I,II_β_I,II_^–1^, *b*_I,II_ = β_I,II_^–1^. Numerical fits, shown in Figure 4a, are in good agreement with the exact positions of CE, obtained by Padé reconstruction of the *S*-matrix element in the complex energy plane. For the *F* + *H*_2_ system, the properties of the CE poles have been extensively studied, e.g., in ref (15), albeit on an older and less accurate Stark-Werner (SW) potential surface (Figure 9 of ref (15)). After accounting for the expected difference between the FXZ and SW PES (for more detail, see Supporting Information B), a comparison in Figure 4b attributes the Regge trajectories I and II in Figure 1d to the well-known in the literature resonances B and A, respectively. The nomenclature was first introduced in ref (5), and subsequently used by other authors. Both A and B are Feshbach resonances, correlated with bound states with support in different regions of the adiabatic potential curve [cf. Figure 3b of ref (13)]. Resonance B is a vdW exit channel resonance, while A, trapped in a deeper well, lies closer to the system’s transition state.^12^ Thus, we find the transition state resonance A, whose tails are clearly visible in Figure 1b,c, to be responsible for the unusual behavior of the state-to-state DCS in Figure 1a.

### II.III Transitions (0,0,0) → (3,1,0) and (0,0,0) → (3,2,0)

**Figure 5:**
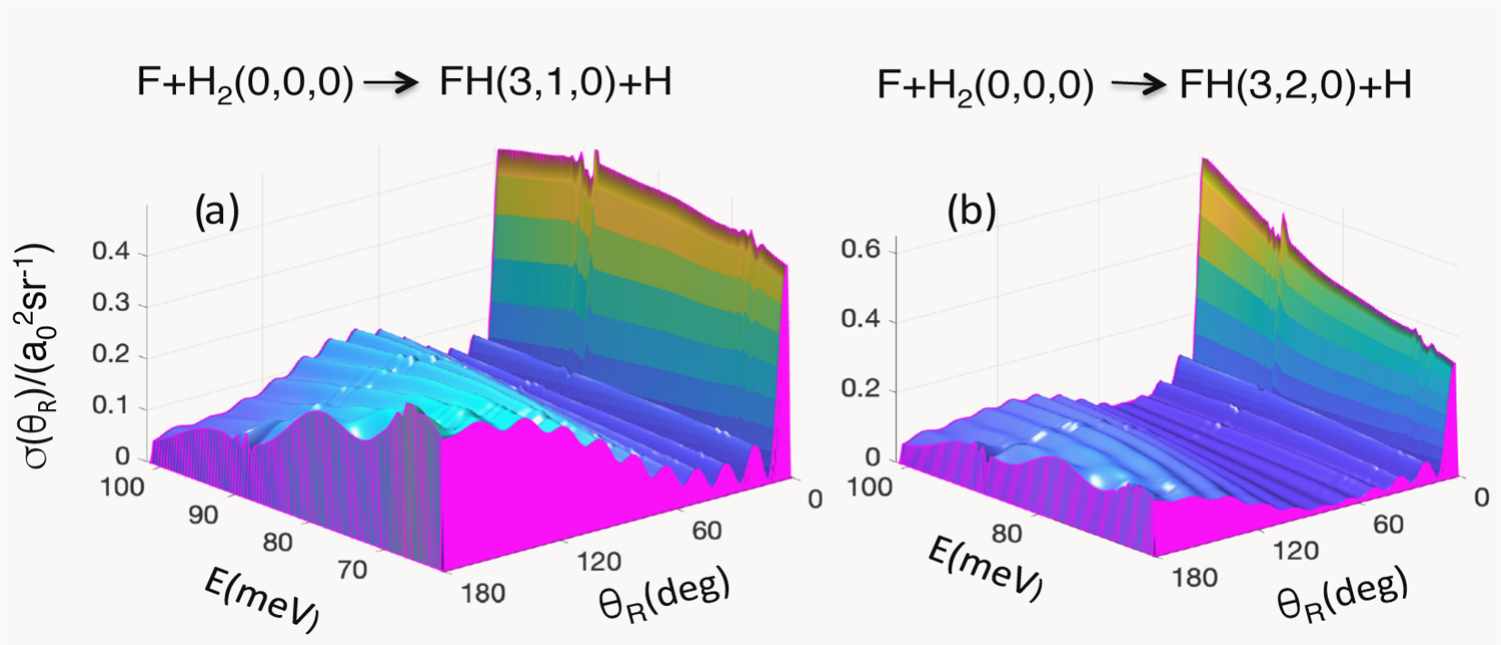
(a) Reactive differential cross section σ_ν_f_←ν_i__(θ_R_) for ν_i_ = (0,0,0) and ν_f_ = (3,1,0) vs θ_R_ and *E*. (b) Same as (a), but for ν_f_ = (3,2,0).

**Figure 6:**
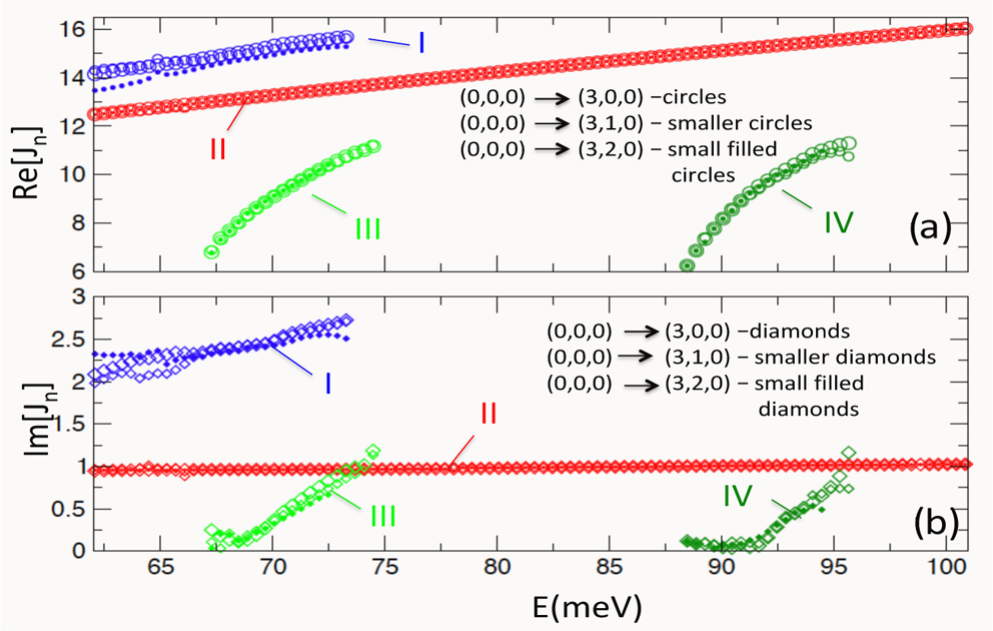
(a) Real parts of the Regge trajectories I, II, III, and IV (circles) for ν_i_ = (0,0,0) and ν_f_ = (3,0,0), (3,1,0), (3,2,0). (b) Imaginary parts of the same trajectories (diamonds).

The shapes of the DCSs of these transitions, shown in Figure 5, are similar to the one in Figure 1a, and can be analyzed in the same manner (see Supporting Information C). We find that all three transitions share the same Regge trajectories, plotted in Figure 6. Such a coincidence is to be expected, as the singularities, whether in the CE or CAM plane, are shared by all matrix elements, which differ only in the magnitude of the corresponding residues. The (0,0,0) → (3,2,0) transition is, however, different in one respect. Its *f̃*- and *g̃*-amplitudes in Figure S3c,d reveal an additional minimum in the 0 ≤ φ ≤ π region across the whole energy range. This feature can be traced back to a *Regge zero*(29) trajectory shown in Figure S4 of the Supporting Information C.

The zero affecting the (0,0,0) → (3,2,0) transition is likely the cause of a somewhat poorer agreement with the rainbow theory in Figure 8c of ref (18). A more detailed analysis of the (0,0,0) → (3,1,0) and (0,0,0) → (3,2,0) transitions will be given elsewhere.

In summary, the same resonance is responsible for the shape of the DCS in Figure 1a as well as in Figure 5a,b.

## III Conclusions and Discussion

A thorough analysis revealed that a single resonance is largely responsible for the unusual behavior of the *F* + *H*_2_(0, 0, 0) → *FH*(3, *j*, 0) reaction in the entire collision energy range 62.09–101.67 meV. This behavior, we recall, involves a pronounced forward scattering maximum, followed by oscillations clearly visible at all scattering angles [cf. Figures 1 and 5]. The resonance has been identified as the transition state resonance A, extensively studied in refs (5,12−16), to which we refer the interested reader. Its lifetime (i.e., the typical time the metastable complex exists prior to breaking up into products), τ = – ℏ/2Im[*E*_*II*_] is found to be rather short, ≈2 × 10^–16^ sec. for 12 ≤ *J* ≤ 17 [cf. Figure 4a]. It is, however, premature to judge the resonance to be too short-lived to produce observable effects in the corresponding state-to-state DCS. More important in this regard is its angular life ϕ = ℏ/2Im[*J*_II_](i.e., the angle by which the complex rotates before breaking up into products), otherwise given by the product of τ with the complex’s angular velocity ω. The latter can be considerable for a light triatomic with a large rotational constant *B* ≡ Re[*E*_*n*_]/*J*(*J*+1), and for the resonance A we find ϕ fairly stable, varying across the chosen energy range from 30.2° to 27.9° [cf. Figure 6b]. However, even this relatively short angular life is proven to be sufficient to produce the interference patterns in the DCS [cf. Figures 1 and 5].

The resonance B, on the other hand, has a similar lifetime [cf. Figure 4a], but a smaller rotational constant. Its rotation is slower, so the decay has little effect on sideway and backward scattering in Figure 3b,c. Still, we found it responsible for about 20% of the forward DCS in the 62–74 meV energy range, as shown in Figure 3a.

A similar behavior is seen in the DCS of the three transitions considered here, *j*_f_ = 0,1,2. In all three cases, we found two other resonances, whose Regge trajectories were labeled III and IV. These, however, have only minor effect on the DCS, since their residues are too small, and the patterns they produced in the DCS are essentially negligible.

Therefore, the three differential cross sections considered here give a fairly clear example of practically a single resonance (A), capable of dramatically changing the nature of reactive angular scattering in the chosen energy range 63–102 meV. (It is remarkable that both resonances *A* and *B* are found for the PES as different as SW and FXZ [cf. Figure 4b].) However, at lower energies, *E* < 62 meV, we expect the resonance B to play a much more important role. We defer the analysis of this case to our future work.

In conclusion, the proposed analysis, facilitated by the DCS_Regge code, can provide important insight into the reaction’s mechanism. This paves the way for studying more complex chemical reactions.

## Data Availability

The code DCS_Regge is available from Mendeley Data (DOI: 10.17632/gf4gm82n6m.1). It is part of the software suite comprising also ICS_Regge—Numerical Regge pole analysis of integral cross sections (DOI:10.17632/xvgmm8my7k.1) and PADE_II—Extracting poles for resonances from numerical scattering data using Padé reconstruction (DOI:10.17632/pt4ynbf5dx.1) codes. Numerical scattering data used in this work is available on request from Dario De Fazio, email: defazio.dario@yahoo.it

## Supporting Information Available

It contains a brief overview of the theory; complex energy poles for different potential energy surfaces; and unfolded amplitudes for the (0,0,0) → (3,1,0) and (0,0,0) → (3,2,0) transitions (PDF)
