# Supplementary material for: A Transition State Resonance Radically Reshapes Angular Distributions of the F + H2 → FH(vf = 3) + H Reaction in the 62–102 meV Energy Range
Source: ACS Phys Chem Au. 2025 Feb 4;5(2):219–26. doi: 10.1021/acsphyschemau.4c00096 (PMC11950844; doi:10.1021/acsphyschemau.4c00096)
Supplement: Supplementary file 1 — pg4c00096_si_001.pdf [file pg4c00096_si_001.pdf]

Supporting Information for: A Transition State Resonance Radically Reshapes Angular Distributions of the $F + H_2 \rightarrow FH(v_f = 3) + H$ Reaction in the $62 - 102$ meV Energy Range

Dmitri Sokolovski[a,b,c,*], Dario De Fazio[d], and Elena Akhmatskaya[b,e]

[a] *Departmento de Química-Física Química-Física, Universidad del País Vasco, UPV/EHU, 48940, Leioa, Spain*

[b] *IKERBASQUE, Basque Foundation for Science, Plaza Euskadi 5, 48009, Bilbao, Spain*

[c] *EHU Quantum Center, Universidad del País Vasco, UPV/EHU, 48940, Leioa, Spain*

[d] *Istituto di Struttura della Materia-Consiglio Nazionale delle Ricerche, 00016 Roma, Italy and*

[e] *Basque Center for Applied Mathematics (BCAM), Alameda de Mazarredo 14, 48009, Bilbao, Spain*

(Dated: December 12, 2024)

A. The unfolded amplitudes In the classical picture, Jacobi vector $\mathbf{R}_{\mathrm{A}\leftarrow\mathrm{BC}}$, drown from the centre of mass of BC to A, rotates in the positive sense around the fixed direction of the total angular momentum $\mathbf{J}$. For zero-helicity transition studied here, both the initial and final directions of $\mathbf{R}_{\mathrm{A}\leftarrow\mathrm{BC}}$, also lie in the plane. The winding angle φ, swept by the projection of $\mathbf{R}_{\mathrm{A}\leftarrow\mathrm{BC}}$ onto the plane, perpendicular to $\mathbf{J}$ is simply related to the reactive scattering angle θ_{R} as shown in Fig.S1a. For $0 < \varphi < \pi$ one has $\theta_{\mathrm{R}} = \pi - \varphi$. However, the symmetry of the problem is such [1], that a rotation by $\varphi = \pi + \theta_{\mathrm{R}}$ also leads to the same scattering angle. Adding multiples of 2π one obtains all winding angles in Eq.(5) of the main manuscript, consistent with the chosen θ_{R}. An angle $\varphi_m(\theta_{\mathrm{R}})$ falls into "nearside" or "farside" category, depending on whether m is even or odd, respectively.

In the body-fixed frame, used in the calculation of the S-matrix element, φ is the variable conjugate to J [2]. For this reason, transformations from J- to φ-representation in Eqs.(3) contain a simple exponential kernel $\exp(i\lambda\varphi)$. The full scattering amplitude is found by "folding back" the "unfolded amplitudes", i.e., by summing with appropriate factors the

*Electronic address: dgsokol15@gmail.com

values of $\tilde{f}(\varphi)$, or $\tilde{g}(\varphi)$, over all φs consistent with the observational angle θ_R [cf. Eqs.(4)-(6)]. The procedure is illustrated schematically in Fig.S1b.

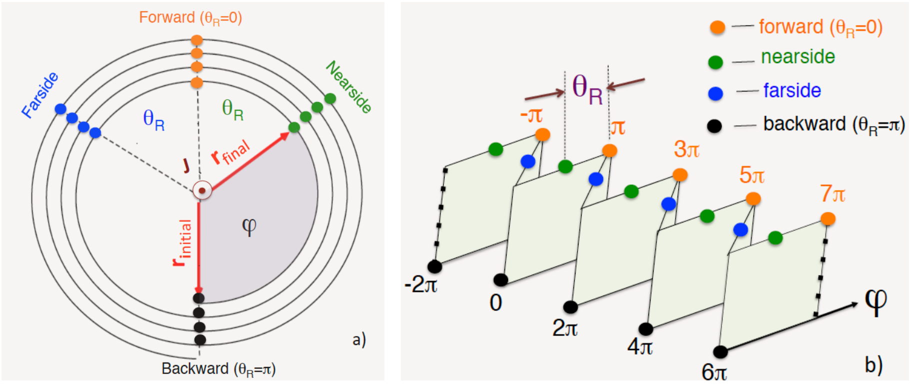

FIG. S1: a) Angle φ between the initial and final directions of a vector $\mathbf{r} = \mathbf{R}_{A\leftarrow BC}/|\mathbf{R}_{A\leftarrow BC}|$. Also shown other winding angles consistent with $0 < \theta_R < \pi$ (green and blue), $\theta_R = 0$ (orange), and $\theta_R = \pi$ (black). b) The values of φ_m over which $\tilde{f}(\varphi)$ is summed in Eq.(4) (green and blue), and the corresponding values for $\tilde{g}(\varphi)$ in Eqs.(6) (orange for $\theta_R = 0$, black for $\theta_R = \pi$).

B. Assignment of Regge resonances. Figure S2 shows the real parts of the complex energy poles of the resonances A and B, obtained by the Q-matrix analysis [3] for the Stark-Werner potential surface (filled triangles). Also shown by the filled circles are the CE poles obtained in the present work by the Padé reconstruction of $S^J_{3,0,0\leftarrow 0,0,0}$ for the FXZ PES. (This can be done with the help of the code `DCS_Regge`, by supplying as input data $S^J_{3,0,0\leftarrow 0,0,0}(E_j)$, for a fixed value of J, and E_j $j = 1, N_j$, chosen on a suitable grid.) The two sets of poles agree if the SW results are lowered by about of 13 meV, and there is a reason for that. The FXZ PES [4] is a relatively recent improvement on the SW PES, developed to reproduce experimentally known exothermicity of the $F + H_2$ reaction with sufficient accuracy. It is also expected to accurately predict the resonance positions, which is why it was used in the present work. Of particular interest to us is the endothermicity of the $HF(v_f = 3)$ threshold energy, which is reduced on the FXZ PES by about 13 meV (see the Table in [5]). It has been shown [3], [6], [7] earlier that the resonance energies are well predicted by the energies of the quasi bound states on the vibrationally adiabatic $FH + H$ potential curves. For this reason, we adjust the SW results shown in Fig.8 of the paper by 13 meV to create Fig.4b. The resulting good agreement, evident in Fig.4b, demonstrates that the CE poles, obtained for the Regge trajectory I (blue circles), correspond to the exit channel resonance B. By the

same token, the CE poles from the Regge trajectory II, can be attributed to the transition state resonance A.

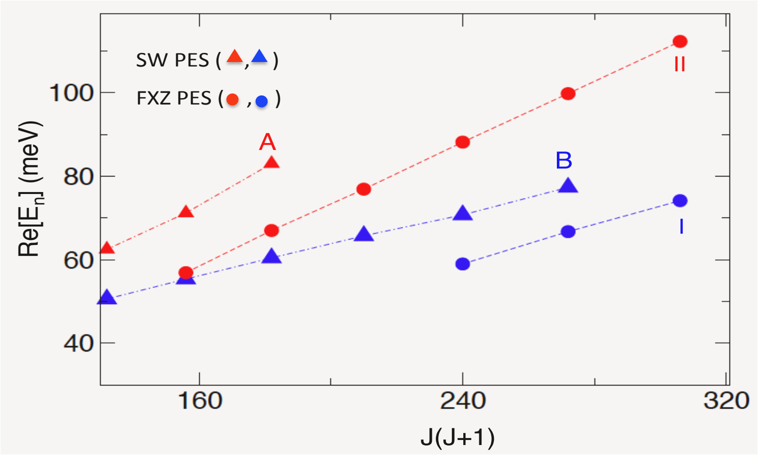

FIG. S2: Comparison of the CE poles, obtained for the Stark-Werner (SW) PES by the Q-matrix method of [3] (triangles) with the poles, obtained for the FXZ PES by the Padé reconstruction (circles, present work).

C. Transitions $(0,0,0) \to (3,1,0)$ *and* $(0,0,0) \to (3,2,0)$. The amplitudes $\tilde{f}(\varphi, E)$ and $\tilde{g}(\varphi, E)$, shown in Fig.S3, exhibit in the region $\varphi \geq \pi$ decaying tails, similar to those seen in Figs.1b and c. As in the case of the $(0,0,0) \to (3,0,0)$ we attribute them to the Regge trajectory II in Fig.6, corresponding to the resonance A of Sect.II *B*. The zeroes of $S_{3,2,0\leftarrow 0,0,0}(J, E)$ in the complex J-plane are shown in Fig.S4. One notes a regular zero trajectory, responsible for the trough in Figs.S3c and d (highlighted). Also clearly visible are the zeroes which accompany Regge trajectories III and IV shown in Fig.6 (note that no zeroes accompany the pole trajectories I and II).

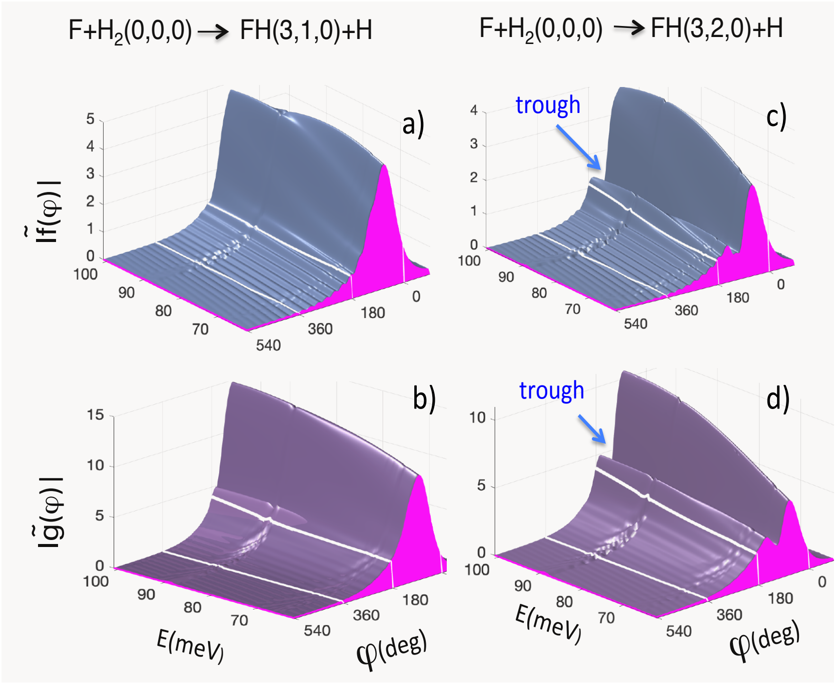

FIG. S3: a) The modulus of the unfolded amplitude $\tilde{f}(\varphi, E)$ for the $(0,0,0) \to (3,1,0)$ transition. b) The modulus of the unfolded amplitude $\tilde{g}(\varphi, E)$ for the $(0,0,0) \to (3,1,0)$ transition. c) Same as (a) but for the $(0,0,0) \to (3,2,0)$ transition. d) Same as (b) but for the $(0,0,0) \to (3,2,0)$ transition.

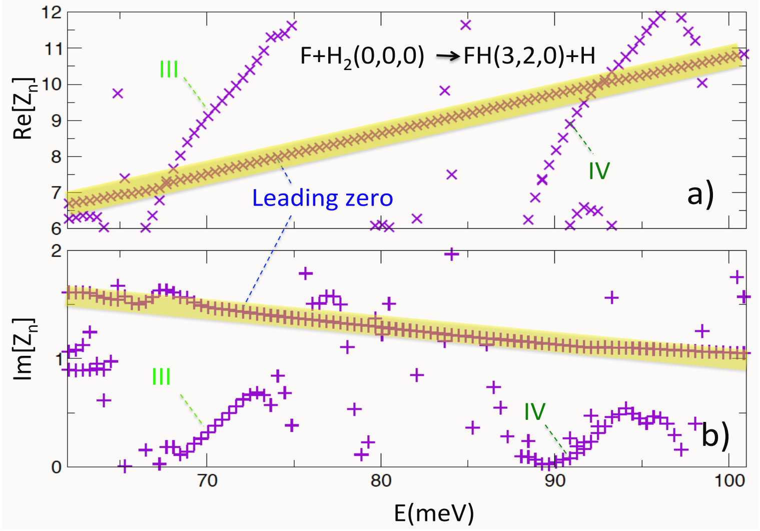

FIG. S4: a) Real parts of the CAM zeroes (crosses) of $S_{3,2,0 \leftarrow 0,0,0}$ vs. E. b) Same as (a) but for the zeroes' imaginary parts.

References

[1] Akhmatskaya, E.; Sokolovski, D. Numerical Regge pole analysis of resonance structures in state-to-state reactive differential cross sections. *Comp. Phys. Comm.* **2022**, *277*, 108370

[2] Sokolovski, D.; Connor, J. N. L. Semiclassical nearside-farside theory for inelastic and reactive atom-diatom collisions. *Chem. Phys. Lett.* **1999**, *305*, 238

[3] Aquilanti, V.; Cavalli, S.; Simoni, A.; Aguilar, A.; Lucas, J. M.; De Fazio, D. Lifetime of reactive scattering resonances: Q-matrix analysis and angular momentum dependence for the $F + H_2$ reaction by t he hyperquantization algorithm. *J. Chem. Phys.* **2004**, *121*, 11675

[4] Qiu, M.; Ren, Z.; Che, L., et al. Observation of Feshbach Resonances in the $F + H_2 \to HF + H$ Reaction. *Science* **2006**, *311*, 1440

[5] Ren, Z., et al. Probing the resonance potential in the F atom reaction with hydrogen deuteride with spectroscopic accuracy. *PNAS* **2008**, *105*, 35

[6] Castillo, J. F.; Manolopoulos, D. E.; Stark, K.; Werner, H.-J. Quantum mechanical angular distributions for the $F + H_2$ reaction. *J. Chem. Phys.* **1996**, *104*, 6531

[7] Aquilanti, V.; Cavalli, S.; De Fazio, D.; Volpi, A.; Aguilar, A.; Gimènez, X.; Lucas, J. M. Exact reaction dynamics by the hyperquantization algorithm: integral and differential cross sections for $F + H_2$, including long-range and spin-orbit effects. *Phys. Chem. Chem. Phys.* **2002**, *2*, 401
